# Adolescent suicide in Ghana: A content analysis of media reports

**DOI:** 10.3402/qhw.v10.27682

**Published:** 2015-05-25

**Authors:** Emmanuel Nii-Boye Quarshie, Joseph Osafo, Charity S. Akotia, Jennifer Peprah

**Affiliations:** Department of Psychology, University of Ghana-Legon, Accra, Ghana

**Keywords:** Adolescent, suicide, Ghana

## Abstract

Adolescent suicide is now a major health concern for many countries. However, there is paucity of systematic studies and lack of official statistics on adolescent suicide in Ghana. Mass media coverage of adolescent suicide (even though crude), at least, may reflect the reality of the phenomenon. With an ecological orientation, this study used qualitative content analysis to analyse the pattern of 44 media reports of adolescent suicide in Ghana from January 2001 through September 2014. Results showed that hanging was the dominant method used. The behaviour usually takes place within or near the adolescent's home environment. The act was often attributed to precursors within the microsystem (family and school) of the deceased. This study serves a seminal function for future empirical studies aimed at deeper examination of the phenomenon in order to inform prevention programmes.

Adolescence—defined in this study as the period between the ages of 10 and 19 years—is a period within the life span when significant physical, cognitive, and socio-emotional changes and challenges occur. Among the myriad challenges that occur during this development period is suicide, which is now a major health concern for many countries (Pompili, Innamorati, Girardi, Tatarelli, & Lester, 2011). Yearly, across the world, it is estimated that 71,000 adolescents die due to suicide and up to 40 times as many adolescents attempt suicide, ranking suicide as the third major cause of death during adolescence (WHO, 2011a, 2014). Significant differences exist in the prevalence and epidemiological pattern of suicide (committed by children, adolescents, and adults) across gender, race, countries, and cultures in the world (Ayyash-Abdo, 2002; Osafo, 2011). However, in Ghana, there are no official statistics on the phenomenon of suicide—as an independent cause or form of death (Eshun, 2003; Knizek, Akotia, & Hjelmeland, 2011). For example, the 2010 Ghana's population and housing census report by the Ghana Statistical Service (GSS, 2013) provides an omnibus statistics of death by suicide, violence, accident, and homicide. The report shows 18,938 deaths recorded and categorized under *deaths by accident*, *violence*, *homicide*, *or suicide* within 12 months preceding the census. Thus, the report merges, rather than isolates the statistics in respect of deaths by accident, violence, homicide, and suicide. However, a recent anecdotal and journalistic report shows that about 1556 people (approximately five people daily) commit suicide annually in Ghana (Citifmonline, 2012).

Similarly, official statistics on adolescent suicide in Ghana is lacking. According to the *children, adolescents*, *and young people in Ghana* segment of the Ghana's 2010 population and housing census report (GSS, 2013), 6467 deaths were recorded among young persons within the age range of 12–19 years, representing 35% of all deaths categorized under *deaths by accident*, *violence*, *homicide*, *or suicide* among young persons (between the ages of 12 and 34 years) within 12 months preceding the census. Hjelmeland et al. (2008) observe that close to half (47%) of university students in Ghana know someone who has attempted suicide and one in five know someone who has killed themselves. Analysis of police-recorded data by Adinkrah (2012) shows that 9.1% of all fatal and non-fatal suicides recorded between 2006 and 2008 involved adolescents (individuals aged between 10 and 19 years). Anecdotal statistics by the Network for Anti-Suicide and Crisis Prevention shows that 531 youngsters (aged between 9 and 19) commit suicide annually in Ghana (Kokutse, 2012). Although these statistics appear staggering and somewhat unreliable, they can be described as the tip of the iceberg because “the fear of social stigma could restrain families and other people from reporting a suicidal person to the police as well as giving a true verdict of the cause of death” (Osafo et al., 2011a, p. 1). The implication is that adolescent suicide has become a daily reality in Ghana (Knizek et al., 2011).

This study thus set out to conduct a situational analysis of adolescent suicide in Ghana through a qualitative content analysis (QCA) of online media news reports of adolescent suicides in Ghana through the lens of the ecological theory of human development (Belsky, 1980; Bronfenbrenner, 1979; Garbriano, 1985). Furthermore, it aimed to add to the search for the epidemiological trends of adolescent suicide in Ghana. Owing to the paucity of systematic studies on adolescent suicide in Ghana, it is hoped that the outcome of this situational analysis will serve a heuristic and seminal function for much broader sociodemographic and epidemiological enquiry into the phenomenon aimed at uncovering the nuances regarding experiences, perceptions, causes, risks, protective factors, prevention, and support systems in the future.

## The ecological approach to the study of adolescent suicide

Suicide literature is replete with several theories (e.g., biological theories, psychological theories, sociological theories, and social psychological theories—such as social learning theory and the family systems theory), which guide and help researchers to structure and provide explanations for their findings. Adolescent suicide is multicausal and may be seen as the consequence of the challenges emerging out of the interplay of biological, psychological, developmental, psychiatric, social, cultural, and family environmental forces at work in the transition from childhood to adulthood (Borowsky, Ireland, & Resnick, 2001; Bridge, Goldstein, & Brent, 2006; Garbriano, 1985). Therefore, a more robust and multidisciplinary model is required to establish a thorough understanding of adolescent suicide (Bridge et al., 2006). In this vein, some adolescent suicide researchers (e.g., Ayyash-Abdo, 2002; Henry, Stephenson, Hanson, & Hargett, 1993) have recommended the application of the human ecological model (Bronfenbrenner, 1977, 1979). The human ecological model appears to be a good fit for the understanding of adolescent suicide because it is a framework that allows for the integration of the array of previous work on adolescent suicide and their families within a single model. As argued by Henry et al. (1993), contrary to the traditional theories of adolescent suicide, the human ecological model is a multidisciplinary approach to understanding adolescent suicide that integrates individual, family, and social system forces, which may be associated with adolescent suicidality within the broader environmental context rather than emphasizing specific risk factors.

Bronfenbrenner (1979) refers to the ecology of human development as involving “the progressive, mutual accommodation between an active, growing human being and the changing properties of the immediate settings in which the developing individual person lives” (p. 21). Bronfenbrenner sees the environment as a series of nested structures, which includes, but transcends, home, school, and the neighbourhood settings within which developing individuals spend their daily lives. Within this model, adolescent suicide is seen as emerging from the adolescent's interactions and interdependencies within hierarchically arranged, multiple-level ecological contexts (Henry et al., 1993). The layers within the ecological model include the individual, microsystem, mesosystem, exosystem, macrosystem, and chronosystems, all in concentric circles (Bronfenbrenner & Morris, 2006). The individuallevel encompasses the individual psychological and personal historical characteristics of the suicidal adolescent (e.g., depression and substance abuse). Within the microsystem, the focus is on the patterns of the roles, activities, and personal relations that adolescents have in the face-to-face settings that form their particular social encounters (e.g., family, school, and peer groups). The layer of the mesosystem concerns the interactions between several microsystems within which children shift between various roles as a result of moving between one microsystem to the other (Bronfenbrenner & Morris, 2006). For an adolescent, this includes the relations among home, school, neighbourhood, peers, and teachers. The exosystem is the social setting that indirectly affects adolescents when they interact with some structures in their microsystem. Adolescents are not directly participating or involved in these social settings, but the process and experiences there affect their development (e.g., formal organizations such as parents’ workplaces, their religious institutions, and health and welfare services in the community). Thus, any resource made available by the exosystem will either work to enrich or impoverish the quality of interactions within the micro- and mesosytems (Harper & Carver, 1999). The macrosystem consists of government; policies; laws and customs of one's culture, subculture, or social class; broad and social ideologies; and values and belief systems. Berk (2006) argues that the priority this system gives to adolescents’ needs affects the support they receive at inner levels of the environment. Thus, opportunity structures and life-course options for the child exist within this system (Muus, Velder, & Porton, 1996). The chronosystem covers the sociohistorical conditions, transitions, and changes in individuals and their environment across time. Thus, it reflects the dynamic environmental transitions, encompassing entries, exits, milestones, and turning points over time in the life of the child (Bronfenbrenner & Morris, 2006).

Although the ecological model is a good fit for the present study, the nature of the data set used (media news reports on adolescent suicide in Ghana) limits the levels of analysis to the individual, microsystem, and macrosystem. The focus of this study is, therefore, on how forces within each of these three layers present and help to identify and understand the trends and risks factors of adolescent suicidal behaviour in Ghana, as reported in the mass media. The application of the ecological approach (which combines the correlates of adolescent suicide in an interactive and additive way) in this study can yield a great advantage. As observed by Ayyash-Abdo (2002), the approach deviates from the tendency to concentrate solely on adolescent personal history (e.g., depression and hopelessness) and additionally shows adolescent suicide as a consequence of an interaction among multiple factors (personal, interpersonal, and sociocultural), which are directly or indirectly connected to adolescents.

## News culture of suicide in Ghana

Act 29, Section 57, of Ghana's criminal code stipulates that, “whoever attempts to commit suicide shall be guilty of a misdemeanour” (Criminal Code of Ghana, Act 29, Section 46 1960). This code, thus, criminalizes attempted suicide in Ghana. Hence, individuals who attempt suicide are subject to arrest and prosecution, and are made to face criminal penalties upon conviction (Adinkrah, 2013; Kahn & Lester, 2013; Knizek, Akotia, & Hjelmeland, 2011; Osafo et al., 2011a). Therefore, like all other forms of crime, suicide is newsworthy (Pirkis, 2009; Romer, Jamieson, & Jamieson, 2006; Sisask & Värnik, 2012). There are over 40 regular newspaper titles, more than 160 FM radio stations, and nearly 10 different free-on-air TV stations in Ghana (Gadzekpo, 2010). This implies that an appreciable number of media avenues exist for information dissemination and discussions of public health issues including suicide. However, it has been observed that, generally, newspapers and other media houses in Ghana are poorly capitalized, poorly staffed, and many are slightly more than cottage industries (Gadzekpo, 2010). The implication is that generating investigative news reports and covering a wide range of specialized subject matters (including complex public health issues such as suicide) is severely hampered. The logic and practice of journalism in Ghana is known to be characterized by fierce competition determined by a mixture of political motives and commercial interests. Thus, the features of competition and commercialization associated with the Ghanaian media appear to, subtly but significantly, influence media houses and journalists as to which events or issues to consider newsworthy.

There is a plethora of evidence that certain types of news coverage of suicide can increase the probable recurrence of the phenomenon (contagion, copycat, or Werther effect) in vulnerable groups—particularly, adolescents (e.g., Gould, Kleinman, Lake, Forman, & Midle, 2014; Niederkrotenthaler et al., 2009; Pirkis, 2009; Pirkis, Burgess, Francis, Blood, & Jolley, 2006; Romer et al., 2006; Sisask & Värnik, 2012; Stack, 2005). Generally, media coverage of suicide cases in Ghana can be described as sensational, explicit, and overly simplistic (Osafo, Hjelmeland, Knizek, & Akotia, 2012); a situation, which deviates from recommended best media practices in the reportage of suicide (Center for Disease Control and Prevention [CDC], 1994). Media coverage of suicides in Ghana often carries sensationalistic headlines and/or prominent placement; they are often allocated front pages, centre spreads, or back pages with images of the suicidal person. Detailed explicit description of the place and method used and identity information of suicidal persons (e.g., name, location, and name of school or workplace) are also detailed and where a suicide note is left, the content is reported, sometimes verbatim. Additionally, quoting or interviewing police, parent, teacher or head of school, neighbour, or first responder about the causes of the suicide prominently features in media coverage of suicide in Ghana. However, strikingly absent from media coverage of suicide in Ghana are the voices of mental and medical health professionals; information on prevention; and education on warning signs, treatment services, and coping skills—a practice described by Osafo et al. (2012) as deficient and narrowed suicide reportage.

Despite these shortcomings, the mass media coverage of suicide, in general, and adolescent suicide, in particular (even though crude), at least, reflects the reality of the phenomenon, given the dearth of scientific research on the phenomenon in Ghana. Not all cases of adolescent suicide get media coverage, though. Online news stories can, however, offer valuable qualitative research data (Schreier, 2012; Sisask & Värnik, 2012). Thus, the Ghanaian mass media is still capable of stimulating discussions and scientific research on adolescent suicide in Ghana and remains an important channel for suicide prevention communication, empowerment, and continuous proactive public psycho-education on suicide.

## Methodology and data source

Ghana is located north of the equator, on the west coast of sub-Saharan Africa. It shares borders to the east and west with Togo and Côte d'Ivoire, respectively. The Gulf of Guinea occupies the south and Burkina Faso shares borders with the north of Ghana. Ghana is largely heterogeneous in terms of language, ethnic, and religious groupings. According to the GSS (2013), approximately 71% of Ghana's population is Christian, 18% is Muslim, 5% adheres to African Traditional religious beliefs, and 6% identifies as belonging to other religious groups or without any religious beliefs. According to the 2010 Population and Housing Census (GSS, 2013), Ghana's population stands at 24,658,823 of which 22.4% represents adolescents (persons between the ages of 10 and 19 years). The proportion of male population classified as adolescents is higher than that for females. The urban–rural variation shows that in the rural areas, 24.4% of the male population is represented by adolescents aged 10–19 years compared to 21.4% of the females. A little less than a quarter of all persons in Ghana are adolescents aged 10–19 years with two in every five persons in Ghana being less than 15 years and almost one in four, an adolescent. Thus, Ghana has a largely youthful population: children, adolescents, and youth constitute a greater proportion of Ghana's population and are exposed to a number of physical, social, mental, and reproductive health risks and challenges (GSS, 2013).

The media sources used for this study are the websites of popular newspapers, FM stations, and some general news agencies in Ghana. The newspapers are *Daily Graphic*, *Daily Guide*, and the *Ghanaian Times*. The FM stations are *Joy FM* and *Peace FM*. The general news agencies are *Ghanaweb* and *Ghana News Agency*. All three selected newspapers (*Daily Graphic*, *Daily Guide*, and the *Ghanaian Times*) are the key newspapers in Ghana (Prah & Yeboah, 2011). *Daily Graphic* (graphic.com.gh) is a state-owned daily newspaper, which mirrors a news culture of covering officialdom as it is read by policymakers and other influential leaders. It is the most widely circulated newspaper in Ghana (Gadzekpo, 2010). *Daily Guide* (dailyguideghana.com) is a privately owned newspaper representing what can be considered as a well-known newspaper in the country (Gadzekpo, 2010). The third newspaper, the *Ghanaian Times* (ghanaiantimes.com.gh) is a state-owned newspaper aimed at business as well as social and political issues and was chosen for this study because it represents what can be described as a specialized newspaper. *Joy FM* (myjoyonline.com) and *Peace FM* (peacefmonline.com) are the most listened to commercial, popular, and leading Ghanaian radio stations in Ghana (Adinkrah, 2014; Prah & Yeboah, 2011). The websites of *Ghanaweb* (ghanaweb.com) and the *Ghana News Agency* (ghananewsagency.org) provide detailed stories about topical news of issues including health; business and economics; politics; science; education; and sports.

The sample for this study was made up of all cases of adolescent suicide in Ghana published on the websites of the selected newspapers, FM stations, and general news sites from January 2001 to September 2014. An archival search of the website of each of the selected media sources was done using keywords such as “suicide,” “adolescent suicide,” “teen suicide,” “youth suicide,” and “student suicide.” This search generated a pool of news reports on suicide—in general—across the specified period. Each news story generated through the search was scanned with the purpose of identifying and separating news reports on adolescent suicidal behaviour from other suicidal cases (e.g., suicides involving younger children and adults), cases of domestic and school accidents, adolescent self-injurious behaviours without suicidal intentions, and other adolescent injury-related deaths (Ohene, Tettey, & Kumoji, 2010). In total, 44 adolescent suicide news stories spanning January 2001 to September 2014 were identified and drawn from the websites of the selected newspapers, FM stations, and news agencies.

### Analysis of data

The 44 media reports on adolescent suicide drawn were subjected to QCA (Burnard, 1996; Graneheim & Lundman, 2004; Schreier, 2012) in an effort to determine the dominant trend and pattern of the phenomenon in terms of prevalence, sociodemographic characteristics, causes, risk factors, and methods used. The QCA proceeded in the four-stage approach suggested by Burnard (1996). At the first stage, each of the authors independently read and re-read all the 44 cases of adolescent suicide drawn from the selected media sites in order to be familiar with the reports and to manually note as many plausible initial “open codes” as possible across the entire data set. The coding was both data-driven (i.e., the coding frames matched the specifics of the data set) and theory-driven—the ecological approach to human development was applied to develop the levels of analysis of coding frames (Bronfenbrenner, 1979; Schreier, 2012). The second stage focused on reading the materials more closely as a research team and agreeing on a set of initial codes we considered very relevant to the data in order to reduce the number of words and phrases so as to produce a manageable list of headings that account for all the data in the materials. Third, we integrated the relevant generated initial open codes that were similar to preliminary codes to aid the search for categories and emerging themes to help explain larger sections of the data. We collated all the preliminary codes and sorted them into meaningful units showing possible themes. At the fourth stage of the analysis, we reviewed and refined the themes and selected extracts, which supported and described the themes. Additionally, we sought to find explanations to the emerged themes and categories in the light of theory, previous related studies, and the general Ghanaian sociocultural context. Generally, to improve validity of the interpretations and findings, codes and themes were thoroughly discussed and agreed upon by all authors before further analyses were done. These cross-validation and group interpretations were to reduce bias and increase the credibility and trustworthiness of the findings (Whittemore, Chase, & Mandle, 2001).

## Findings

### Trend of adolescent suicidal behaviour

**Figure 1:**
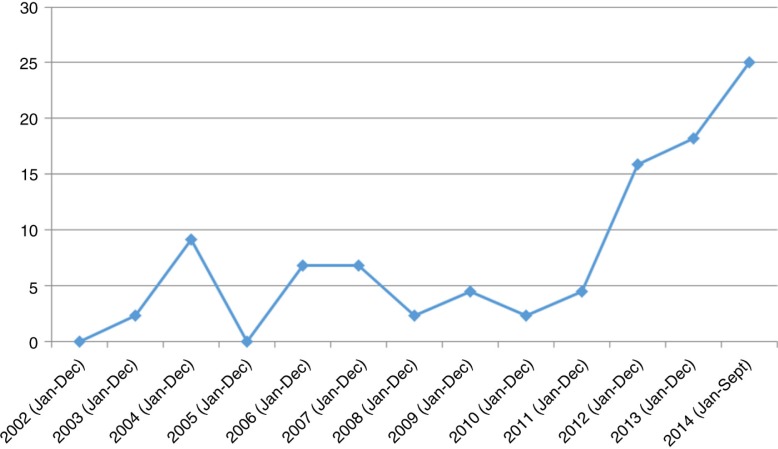
Curve of cases of adolescent suicide from January 2001 through September 2014.

Over the study period from January 2001 through September 2014, a total of 44 adolescent suicides were reported on the websites of the selected media sources. Of this number, 40 cases, representing 90.9%, were completed suicides and 4 (9.1%) were attempted suicides. As shown in Figure 1, the incidence of adolescent suicide assumed an upward spiral increase in 2012 with more completed than attempted suicides. Seven cases were reported in 2012, representing 15.9% of all the 44 cases reported over the study period. Of the seven cases, six were completed suicides and one was an attempted case. In 2013, eight cases (representing 18.2%), all of which were completed suicides, were reported. As of the end of September 2014, 11 cases (representing 25% of all 44 cases) had been reported. Similarly, all the adolescents involved in these 11 cases died through the act (completed suicides).

### Important demographics

The mean age of the girls was 15.4 years, whereas that of the boys was 16 years. Of the identified and selected cases of adolescent suicides over the study period, more boys than girls (54.5%: 31.8%) attempted (6.8%: 2.3%) and completed (47.7%: 29.5%) the act. The sex of six completed cases (13.6%) was not reported. Regarding the method used to complete or attempt the act, hanging was the most common (72.7%), followed by poisoning (11.3%) and slitting (2.3%). The methods used in six cases (13.6%) were not reported. Of the girl suicides (31.8%), 25% used hanging, 4.5% poisoned themselves, and the method used by 2.3% was not reported. Among the boys (54.5%), 45.4% hanged themselves, 6.8% used poisoning, and 2.3% used slitting. Thus, there was no significant difference between boys and girls in the methods used to complete or attempt the act (Table I). Thus, the majority of the fatalities were through hanging and poisoning. This diverges slightly from the firearm means among adults reported by Adinkrah (2012). Those who completed or attempted the act through hanging made use of rope, cloth, wire, or sponge (nylon net). Those who adopted poisoning ingested weedicides or pesticides; and slitting involved the use of a nail or other sharp objects.

**Table I:** Gender and method used.

	Method used to attempt or commit act				
	
Gender	Hanging	Poisoning	Slitting	MNR	Total
Male	45.4% (20)	6.8% (3)	2.3% (1)	0.0% (0)	54.5% (24)
Female	25% (11)	4.5% (2)	0.0% (0)	2.3% (1)	31.8% (14)
GNR	2.3% (1)	0.0% (0)	0.0% (0)	11.3% (5)	13.6% (6)
Total	72.7% (32)	11.3% (5)	2.3% (1)	13.6% (6)	100% (44)

MNR: method used not reported; GNR: gender not reported.

Analysis of the identified and selected adolescent suicide news reports further showed that the home was the most commonly used location (61.3%) for suicidal behaviour among adolescents. Farms and uncompleted or abandoned buildings near the home or school were also found to be places where adolescent suicidal behaviours occurred (Table II). Typical instances of the act at home and in uncompleted or abandoned buildings involved hanging from a rafter of ceiling in bedroom, sitting room, bathroom, or suicide's own room. On farms, the suicide was hanging from a branch of a tree. Adolescent suicides who poisoned themselves were commonly found at home.

**Table II:** Place of act.

	Place						
	
Suicide	Home	School	Farm	Uncompleted building	Police cell	PNR	Total
Attempted	2.3% (1)	2.3% (1)	2.3% (1)	0.0% (0)	2.3% (1)	0.0% (0)	9.1% (4)
Completed	59.0% (26)	4.5% (2)	4.5% (2)	6.8% (3)	0.0% (0)	15.9% (7)	90.9% (40)
Total	61.3% (27)	6.8% (3)	6.8% (3)	6.8% (3)	2.3% (1)	15.9% (7)	100% (44)

PNR: place of the act not reported.

On the educational background of adolescent suicides, more than half (63.6%) of all the media reported cases across the study period involved adolescents in school. Of this percentage, 15.9% were in primary school, 22.7% were in junior high school (JHS), and 25% were adolescents in senior high school (SHS). Overall, 4.5% of the reported cases involved dropout adolescents (Table III). The educational backgrounds of 14 (31.8%) were not reported. These staggering statistics appear to suggest that the school setting presents some pathways or risk factors to adolescent suicide in Ghana.

**Table III:** Educational background.

Suicide	Primary school	Junior high school	Senior high school	Drop Out	ENR	Total
Attempted	0.0% (0)	2.3% (1)	0.0% (0)	0.0% (0)	6.8% (3)	9.1% (4)
Completed	15.9% (7)	20.4% (9)	25% (11)	4.5% (2)	25% (11)	90.9% (40)
Total	15.9% (7)	22.7% (10)	25% (11)	4.5% (2)	31.8% (14)	100% (44)

ENR: educational background not reported.

Adolescent suicide occurred irrespective of their living situation—whether the adolescents were living with both parents, single parent, extended relations, or living alone. Overall, 20.4% of the adolescent suicides were living with both parents, 38.6% were living with a single parent, and 11.3% were living with (at least an) extended relation (Table IV). The living situation of 25% of the adolescent suicides was not reported. It is, thus, evident that living with a single parent is a suicidal risk factor for adolescents. Another notable observation evident in Table IV is that 70.3% of the reported cases involved adolescents who were living with their parents (single or both) or extended relations. This makes one wonder as to whether parents and significant others living with adolescents observed any warning signs of suicide among their adolescent wards.

**Table IV:** Living situation of adolescent before suicide.

	Living (with)						
	
Suicide	Both parents	Single parent	Extended relation(s)	Alone	Police cell	LNR	Total
Attempted	2.3% (1)	4.5% (2)	0.0% (0)	0.0% (0)	2.3% (1)	0.0% (0)	9.1% (4)
Completed	18.1% (8)	34% (15)	11.3% (5)	2.3% (1)	0.0% (0)	25% (11)	90.9% (40)
Total	20.4% (9)	38.6% (17)	11.3% (5)	2.3% (1)	2.3% (1)	25% (11)	100% (44)

LNR: living situation not reported.

About 34% of all the adolescents who engaged in the reported suicidal behaviours showed signs of their intention to complete or attempt the act (Table V). These signs were in the form of directly observable changes (11.3%), verbal threat to engage in the act (20.4%), and previous attempt of suicide (2.3%). Another notable development shown in Table V is that parents and some significant others reported that adolescent suicides (*n*=22) did not show any sign of suicidal crisis. However, even adolescents who issued verbal suicidal threats (*n*=9) and those who showed clear behavioural changes (*n*=4) all died. It is a plausible conclusion then that parents are not paying attention to suicidal threats and this might reflect the reported negative and trivial attitudes towards suicidal behaviour in Ghana (e.g., Osafo et al., 2011b).

**Table V:** Harbinger of adolescent suicide.

Suicide	Behavioural change	Threat	Previous suicide attempt	No observable sign of suicidal intention	SNR	Total
Attempted	2.2% (1)	0.0% (0)	0.0% (0)	6.8% (3)	0.0% (0)	9.1% (4)
Completed	9.1% (4)	20.4% (9)	2.3% (1)	43.1% (19)	15.9% (7)	90.9% (40)
Total	11.3% (5)	20.4% (9)	2.3% (1)	50% (22)	15.9% (7)	100% (44)

Behavioural change: depressed, unusual behaviour, inactivity, desperate to kill self; Threat: victim threatened suicide to significant other (parents, peers, etc.) but threat ignored; SNR: sign of suicidal intention not reported.

### Narratives of motivation

Motivation for adolescent suicide refers to the reasons given for the adolescent suicidal behaviour (Bridge et al., 2006). In this study, the search for the motivation covered both *assigned* and *confessed* reasons, which accompanied the news reports of the phenomenon on the websites of the selected media sources. The assigned reasons were those reasons suggested by the police, significant others (e.g., parents, peers, and neighbours), or the media source for an adolescent suicidal behaviour. Confessed reasons were those reasons given by the suicidal adolescent. These confessions were in the form of suicide note, confession before death (especially in cases of self-poisoning), or the adolescent's confession following an attempted suicide. The narratives of motivation for adolescent suicidal behaviours were organized around five subthemes: *psychological factors*, *conflictual relationships*, *loss of significant other*, *poor school work*, *and socio-economic factors*.

*Psychological factors*: Some adolescents (13.5%) engaged in the suicidal behaviour because of psychological distress and unwanted pregnancy. Some of the adolescents (9.0%) were reported to have shown signs of psychological distress prior to their suicidal behaviour. For instance,

Mother of the suicide (boy, 19 years) told the police that on the fateful day, she returned from the market at about 7:30 pm and met her son moody. She questioned him, but he remained dumb-founded only to wake up the following morning to see the lifeless body of her son. (reason assigned by significant other)

Similarly, it was reported in another case that,

The deceased (girl, 17 years), who was on vacation, appeared very depressed and was showing an unusual behaviour at home. But when her mother inquired to know what was wrong with her, she did not open up. Friends of the suicide told the mother that she was perhaps showing signs of depression because she could not write her terminal examinations. (reason assigned by police)

In both narratives, the adolescents were perceived to have experienced some form of depression. This finding appears to be consistent with the observation by WHO (2011b) that psychological distress and depression is the single largest contributor to the worldwide burden of mental health problems and diseases for people aged 15–19.

*Unwanted pregnancy* was found to be a notable reason for suicidal behaviours among boys who had impregnated a girl. This reason accounted for 4.5% of all the cases over the period. For example, it was reported in one case that,

The deceased (boy, 17 years) killed himself because the families of two girls, on separate occasions, informed him that he had impregnated their daughters and that he was going to be a father in some few months. (reason assigned by police)

In another case, it was mentioned that, the deceased (boy, 17 years) had impregnated a 15-year old girl” (reason assigned by significant other).

The contemporary society of Ghana expects children and adolescents to remain in school. The patriarchal nature of the society requires boys and young men to go through school or vocational training, secure good employment, and be able to pay the bride price of their potential wives before beginning to raise their own children. Pregnancy out of wedlock is generally abhorred by the society and is particularly a burden for the boy or man responsible, as he is usually required to take up the financial responsibility of caring for the pregnant woman (Sarpong, 2006). Thus, children, and for that matter adolescents, should not rush into adulthood by doing things that are considered preserve of adults, otherwise they would be punished like adults (Gyekye, 2003). It is a common social fact in Ghana that teen pregnancy brings the respective families of the boy and girl involved into moral disrepute. For adolescent boys, this can be an overwhelming challenge as indicated in the narratives above. It is possible that these boys were still in school, unemployed, and were not fully prepared to take up the roles of husbands and fatherhood.

*Conflictual relationships* covered elements related to parent–adolescent communication and interaction patterns difficulties within the home environment, which lead adolescents to be suicidal. Accusations and scolding, parental disapproval, and maltreatment and corporal punishment were identified as characterizing such conflictual relationships. Accusations and scolding involved parents (or guardians of adolescents) being hostile to and levelling accusations against their adolescent children. Some adolescents (18.1%) were engaged in suicidal behaviour because they were either accused of and/or scolded by their parents or guardians for a behaviour the parents or guardians considered to be wrong. For instance, in one completed suicide report, the adolescent (girl, 19 years) narrated in her suicide note as follows:

Mother says I had an abortion and when I dress to school, I go about having sex with men. This is not true. I know that my own sister hates me because of this and mother as well, so I am ending it all … I can never in my life kill even an insect, let alone an abortion. (extract from suicide note)

In another instance, the police coroner reported that, “the suicide (female, 10 years) reportedly killed herself after she was scolded by her mother for misbehaving.”

Children in Ghana are socialized to be obedient to their parents and to respect their elders. They are also exhorted to submit to parental control, advice, or authority (Gyekye, 2003). However, the parents of the adolescents in the narratives above seemed to have exercised their right to parental control but did not demonstrate respect for the views of their adolescent children on the purported misbehaviours.

Some adolescents (18.1%) were engaged in suicidal behaviour because their parents disapproved of their peer friendships and relationships. In one case, a media source reported that, “distraught over persistent calls by his mother to end a relationship with a woman older than him, a third-year student (aged, 19 years) of a senior high school has hanged himself.” In another case,

A neighbour reported that the deceased (female, 14 years) threatened to take her life if her mother refused to hand over her phone. He explained that her mother seized the phone because she suspected the deceased of communicating with boys. (reason assigned by neighbour)

The behaviour of the parents involved in the above narratives find explanation in the Akan maxim (which is also subscribed to by many other ethnic groups in Ghana) that, “one has not been an elder before but one has been a child—before” (Gyekye, 2003, p. 86). In other words, because children are inexperienced in life, their parents or elders (who have rich life experiences) do not only know what is good for them but are the best people to choose what is good for them. Thus, as indicated in the above narratives, a parent's disapproval of an adolescent's choice of friends, even if based on mere suspicion, means that friendship must be dissolved by the adolescent child. This socio-cultural authority and position of “I know what is best for you” of parents that underpin parental disapproval of adolescent friendships appear to serve as a precursor to adolescent suicide.

Maltreatment and corporal punishment was assigned as the reason for 11.3% of all the reported cases of adolescent suicides across the study period. In all the cases attributed to this factor, the suicidal adolescent was maltreated and given corporal punishment by a parent, guardian, or a significant other. For example, it was mentioned in a completed suicide news report that:

The deceased (boy, 15 years) allegedly stole GH¢4.00 from his grandfather and was given some lashes by his uncle while the auntie threatened to report the matter to his school authorities for further punishment. The next day, the adolescent refused to go to school for fear of being punished by authorities. He committed suicide later in the day. (reason assigned by police)

In a case of an attempted suicide, the adolescent told the police investigating the case that, “he (boy, 16 years) was living with his father and wanted to take his life because his father maltreated him and did not cater for him” (reason assigned by police).

The use of corporal punishment and other forms of maltreatments (usually, misconstrued as helping to correct wrong behaviours) still characterize the parent–adolescent interaction in Ghana (Ananga, 2011). Traditionally, in the parental role of raising children, parents are expected to discipline their children and be firm in dealing with them (Gyekye, 2003). However, as indicated in the narratives above, some parents unnecessarily resort to the use of some dehumanizing measures (such as flogging, starving, refusing to pay school fees, etc.) as means of disciplining their adolescent children. The use of dialogue and parent–adolescent conference hardly characterize the resolution of parent–adolescent conflict in Ghana. Another interesting revelation in the former narrative is that one of the reasons why the adolescent committed suicide was because of the *fear of being punished in school*. Corporal punishment and other forms of inhumane treatments of students still exist in many basic and second cycle schools in Ghana (Agbenyega, 2006; Ananga, 2011; Lewin & Akyeampong, 2009). The mode of meting out these punishments in the schools can be psychologically disturbing and traumatizing for children and teens because the culprit is usually given the punishments in the presence of an entire class or school with peers and mates sometimes required to lampoon the culprit in the process. This is a situation which has the potential of making the punished adolescent develop a sense of shame and dishonour, a phenomenon which characterizes male suicidal behaviour in Ghana (Adinkrah, 2013).

Some of the adolescents (13.5%) took to suicide because they had lost a significant other through break-up or death of a parent. All the cases of adolescent suicide (9.0%) for which break-up was cited as the reason were reported entirely by girls who had been jilted by their boyfriends. For instance, in one adolescent completed suicide case, *the adolescent (girl*, *17 years) confessed to her mother (few moments to her death while on the way to hospital) that she had drunk some weedicide because her boyfriend, who lives in nearby town, had jilted her* (reason assigned by significant other).

Death of significant other accounted for (4.5%) of all the cases. It was particularly cited where the adolescent had lost a (single) parent to death. For example:

The deceased (boy, 19 years) informed his friends and close associates that he wanted to ‘travel’ or commit suicide due to information of his father's death … The deceased attempted ending his life once but was stopped by his friends and dormitory mates and a report was made to the Senior Housemaster of the school who took him to the school chaplain for counselling. Four days later, the body of the deceased, was found hanging on one of the teak trees in the schools’ teak plantation. (reason assigned by police)

Poor academic performance was notably identified as the reason for adolescent suicidal behaviours among boys (6.8%).

The deceased (boy, 19 years) obtained grades in the just ended West African Secondary School Certificate Examinations (WASSCE) which fell short of the required eight ‘As’. He took the action because his friends had eight ‘As’ which qualified them for admission to the medical school. (reason assigned by police)

In another case, the deceased (boy, 18 years) dropped out of a public school about 6 years ago for poor academic performance and enrolled in a private school but dropped out for the same reason (reason assigned by police).

Socio-economic factors such as parental poverty and the cultural practice of child marriage were also found to be reasons for adolescent suicidal behaviour. Parents’ (especially single parents’) inability to pay the school fees of their adolescent children was mentioned as the motivation for adolescent suicidal behaviour. All the identified cases (9.0%) involved only adolescent girls living with single mothers.

The deceased (girl, in SHS 2nd year) living with a single mother did not go to school on Monday because she owed school fees. The mother asked her in the morning to go to school with the promise of looking for a loan to settle the school fees of GH¢190, but the daughter committed suicide later in the day. (reason assigned by police)

The deceased (girl, 17 years) committed suicide over what was believed to be her frustration over her mother's inability to pay her school fees. (reason assigned by police)

The cultural practice of child marriage was identified as the reason for suicidal behaviour among some adolescent girls (4.5%).

A 14-year-old girl has been rescued from hanging herself, after escaping from her parents who wanted to force her into marriage (reason assigned by media source). In another news report, the deceased (a 16-year-old JHS drop-out) has committed suicide because she was forced into marriage against her wishes (reason assigned by police). The cultural practice of giving an underage girl child into marriage with an adult man (child betrothal) was very common in traditional Ghana. However, the practice still appears to have currency in some communities (in the northern) parts of Ghana although the influence of education (i.e., formal classroom education) has led to a general incessant decline in the practice among the various ethnic groups in the country (Nukunya, 2003; Serra, 2009). It is now common knowledge that the practice, apart from it being a right and child abuse issue, prevents the girl child from growing and developing her full potential even as an adult. Thus, some girls who are unable to find better ways of escaping the practice resort to suicide.

## Discussion

The present study provides a situational and seminal analysis of the phenomenon of adolescent suicide in Ghana through the analysis of media news reports of 44 adolescent suicide cases from January 2001 through September 2014. Analysis of demographic characteristics and trends showed that adolescent suicide has become an everyday reality in Ghana characterized by an upward spiral increase with high fatality rate. Furthermore, the suicidal adolescent was found to be in school, living with (at least) a parent or extended relation, and typically commits the act by self-hanging at home or within its immediate environs. More adolescent boys, than girls, attempted and completed the act. The search for the motivation behind the act among adolescents showed the following notable factors: *psychological factors*, *conflictual relationships*, *loss of significant other*, *poor school work*, *and socio-economic factors*.

The narratives of motivation behind the act fit well with the ecological model of human development (Ayyash-Abdo, 2002; Bronfenbrenner & Morris, 2006; Henry et al., 1993). The psychological motivations can be placed at the *individual level* as the categories identified represent personal characteristics and historical events of the individual adolescent. Thus, in the present study some of the adolescents became suicidal by virtue of their individual psychological and personal historical characteristics of distress and unwanted pregnancy. More than half of the adolescents were students. Recent studies have reported high incidence of psychological distress, depression, and anxiety disorders among students in Ghana (Canavan et al., 2013; Oppong & Andoh-Arthur, 2014).

The reasons of conflictual relationship and loss of significant other can all be placed within the concentric circle of the microsystem layer. The specific factors identified are those found within the home and school settings. These factors influence adolescent suicidality because the patterns of roles, activities, and personal relations of adolescents in this layer involve face-to-face interactions and direct social encounters. It is evident from the present study that microsystem reasons account for more cases of adolescent suicidal behaviours than any other level of analysis.

Socio-economic factors (associated with poverty and the cultural practice of child marriage) can be found within the macrosystem. Ghana is a lower middle income level country with some communities and families struggling to exit abject poverty. The situation is even much difficult for single parents raising school-going children. Quarshie (2011) found that macrosystem factors such as poverty and the cultural practice of child betrothal (child marriage) in Ghana are notable precursors to children and adolescents taking to street living and other life styles, which are potentially harmful to their psychosocial well-being.

## Conclusion and implications

It can be concluded that the observed increase in adolescent suicide in Ghana in recent years may be attributable to motivational forces and risk factors found, largely, within the microsystem and the macrosystem—forces that are beyond the individual adolescent. A few recommendations can be made based on the findings of this study for prevention and risk reduction programmes, and reporting adolescent suicide cases in the media. First, the present study has shown that the majority of suicidal adolescents are students, hence teacher gatekeeper programmes on screening for at-risk adolescents in school can be instituted. Gatekeeper training programmes have been found to be very important in any suicide-prevention programme and many early intervention programmes have used schools as the main setting for identifying and/or intervening with adolescents (Pompili et al., 2011). Teachers can be trained on the use of screening tools to periodically assess students on depression (and other forms of psychological distress), current and previous suicidal ideation and attempts, and other risk factors of suicide. Students found to be showing clinically significant symptoms of the risk factors can be referred for professional attention.

Curriculum-based programmes on education and awareness about suicide can also be introduced in schools in Ghana. Health education programmes about suicide in schools have been found to generally help dispel the myths, increase the correctness of knowledge (including signs) of adolescent suicide, and encourage the attitude of help-seeking when necessary (Pompili et al., 2011). Any such curriculum approach should focus on destigmatizing suicide and identifying suicide as a complicated psychological reaction to a number of overwhelming factors (Lasear, Roggenbaum, & Blase, 2003).

Crisis service providers including psychologists, social workers, and other mental health professionals can collaborate with schools to educate and train parents to enhance parenting skills, knowledge, and confidence. This collaborative gatekeeper training of parents has been found to be effective in adolescent suicide prevention (Pompili et al., 2011). In this strategy, parents are provided with information regarding warning signs, risk factors, protective factors, community resources, and what to do following adolescent suicidal crisis within the home environment. Again, this effort helps parents to improve their communication patterns with their adolescents and encourages parents to move from negative to positive interpretations of adolescent behaviours (Pompili et al., 2011).

Finally, the upward increase in the incidence of adolescent suicide in Ghana calls for the education of members within the wider community on the phenomenon. As suggested by others (e.g., Osafo, Akotia, Andoh-Arthur, & Quarshie, in press), community psychologists, social workers, and community mental health workers have to intensify community psycho-education to reduce the public stigma and the attitude of triviality towards suicide in Ghana. On reporting adolescent suicide in the media, the police, public officials, and journalists should carefully explain that the final precipitating event was not the only cause of a given suicide. Most persons who have committed suicide have had a history of problems that may not have been acknowledged during the acute aftermath of the suicide (CDC, 1994). Therefore, journalists and other role players in the Ghanaian media should be educated and trained on reporting suicide as the present style of reporting (described earlier) can, potentially, have a copycat effect on vulnerable adolescents. An investigation is already underway by the authors of this paper in an attempt to establish any evidence of copycat effect of the present reporting style of suicide by the media in Ghana to warrant any such training.

## Limitations

Media reports (including online news stories) of adolescent suicide do not represent official reports or national authoritative data, partly because they are fraught with reporting biases, exaggeration, and sensationalism (Sisask & Värnik, 2012). Thus, a critical look at the present study shows a few limitations and methodological challenges, which may undermine the generalizability of the findings. First, not all cases of adolescent suicide get media attention (Osafo et al., 2012), a factor which might have led the authors to underestimate the actual trend and scope of the incidence of adolescent suicide in Ghana. Second, journalists fall on police verbal report at the scene of the suicide to feed their news reports. Attempted suicide is a crime in Ghana (Adinkrah, 2013; Osafo et al., 2011a), and this fact can limit the amount of causal explanations a journalist can obtain from the coroner at a suicide scene as too much information divulged can “inhibit” police investigation to unravel the “true” cause of the suicide. Third, the methodology utilized did not allow the researchers to adequately explore the protective factors associated with the phenomenon, at least from the perspective of attempters in general. The narratives of motivation for the act provided in this study were largely suggested or assigned by the police and significant others. Despite these limitations, the study provides useful insights and a seminal point of departure for a broader rigorous systematic enquiry into adolescent suicide to establish the trends, and to understand adolescent suicidality risk factors and other correlates to help build an empirical basis for intervention, risk reduction programmes, and prevention strategies of the phenomenon in Ghana.
